# Lysine demethylase 5D promotes CHEK1 inhibitor sensitivity through p38-mediated cyclooxygenase-2 expression in castration-resistant prostate cancer cells

**DOI:** 10.1016/j.jpet.2025.103769

**Published:** 2025-11-04

**Authors:** Wenxiao Zheng, Shichen Li, Raymond E. West, Ella R. Donahue, Thomas D. Nolin, Song Li, Qiming Jane Wang

**Affiliations:** 1 Department of Pharmacology and Chemical Biology, University of Pittsburgh, Pittsburgh, Pennsylvania; 2 Center for Pharmacogenetics, Department of Pharmaceutical Sciences, University of Pittsburgh School of Pharmacy, Pittsburgh, Pennsylvania; 3 Small Molecule Biomarker Core, University of Pittsburgh School of Pharmacy, Pittsburgh, Pennsylvania

**Keywords:** Cell cycle checkpoint kinase, Prostate cancer, Epigenetic regulator, Protein kinase inhibitor, Drug sensitivity

## Abstract

CHEK1 (CHK1) is a key regulator of the G2/M checkpoint and DNA damage response. Although CHK1 inhibitors (CHK1is) show promise in multiple clinical trials, their further advancement is hampered by the lack of reliable predictive biomarkers. Our previous study demonstrated a nearly 20-fold difference in the sensitivity to a clinical-stage CHK1i SRA737 in prostate cancer (PC) cells. Through bioinformatics analysis, an epigenetic regulator, lysine demethylase 5D (KDM5D), was identified as a potential mediator of differential responses to SRA737. Gain- or loss-of-function studies were performed to investigate how altered KDM5D expression affects CHK1i sensitivity and the underlying mechanisms. Our data demonstrated that higher KDM5D expressions correlated with greater sensitivity to CHK1is in PC cells. In patients with castration-resistant PC (CRPC), a high KDM5D score predicted a better patient response to CHK1i. Knockdown of KDM5D in SRA737-sensitive KDM5D-expressing cells caused resistance to SRA737. Correspondingly, a higher sensitivity to SRA737 was observed in a docetaxel-resistant CRPC cell line with elevated KDM5D, and silencing KDM5D caused resistance to this inhibitor. Mechanistically, depletion of KDM5D activated p38 and induced cyclooxygenase-2 (COX-2) and ATP-binding cassette transporter expression. Inhibition of p38 or COX-2 partially reversed the resistance to CHK1i induced by KDM5D knockdown. Additionally, silencing of p38 increased KDM5D protein expression, indicating a negative feedback loop that may serve to maintain a homeostatic balance between the 2 genes. These data support a key role for KDM5D in modulating CHK1i sensitivity through a novel p38/COX-2 prosurvival pathway in PC cells, with potential predictive value for patients with CRPC receiving these anticancer agents.

**Significance Statement:**

This study demonstrated an important role of an epigenetic regulator KDM5D in regulating CHK1 inhibitor sensitivity via a p38/COX-2-mediated prosurvival pathway in certain castration- or drug-resistant PC cells. Our results indicate that PC cells expressing KDM5D may be more sensitive to targeted inhibition of CHK1 kinase, highlighting the potential predictive value of this gene for CHK1-targeted therapies in PC.

## 1 Introduction

CHEK1 (CHK1) is crucial for cell cycle regulation and DNA damage response. Prostate cancer (PC), especially late-stage castration-resistant (CRPC) and neuroendocrine prostate cancer (NEPC), often exhibits frequent genomic alterations in tumor suppressor genes such as TP53, RB1, and PTEN. These alterations abolish the G1 checkpoint and render tumor cells particularly vulnerable to therapies targeting the G2/M checkpoint and DNA damage response.^1^ Consequently, CHK1, a critical regulator of the G2/M checkpoint, has emerged as a compelling therapeutic target in these cancers.^2^, ^3^, ^4^

CHK1 inhibitors (CHK1is) have shown encouraging results in preclinical and early clinical studies over the past few decades. However, their translation to clinical success has been hampered by several significant challenges. One of the main obstacles is the heterogeneous tumor response to CHK1is, which frequently results in suboptimal outcomes in broader patient cohorts, underscoring the urgent need for reliable predictive biomarkers to identify patients who are most likely to benefit from this targeted therapy. SRA737 is a newer-generation, highly selective, and orally available CHK1i with >1000-fold selectivity against CHK2.^5^^,^^6^ SRA737 has shown favorable safety and efficacy profiles when combined with gemcitabine in preclinical non–small cell lung cancer and MYC-driven B-cell lymphoma mouse models.^5^ Recent reports from 2 clinical trials on SRA737 in advanced cancers (NCT02797964 and NCT02797977) revealed unique attributes of this drug, including lower myelotoxicity and higher but manageable gastrointestinal toxicities that differ from other CHK1is examined in the clinic.^7^^,^^8^ Although no complete or partial Response Evaluation Criteria in Solid Tumors responses were observed in 1 trial that included 13 patients with metastatic CRPC, 8 of 13 (61.5%) patients with metastatic CRPC showed stable disease after 4 therapy cycles, demonstrating the need to improve efficacy and develop better strategies to stratify patients receiving CHK1is.

Lysine demethylase 5D (*KDM5D/JARID1D/SMCY*) is a male-specific histone-modifying enzyme encoded on the Y chromosome that demethylates di- and tri-methylated forms of lysine 4 in histone H3 (H3K4me3 and H3K4me2), generally resulting in the transcriptional repression of certain genes.^9^ KDM5D, together with KDM5A, KDM5B, and KDM5C, belongs to the KDM5 family and has been implicated in various cancers and in the regulation of oncogenes and tumor suppressor genes.^10^ KDM5D’s tumor suppressive activity has been demonstrated in multiple cancers.^9^^,^^11^^,^^12^ In PC, *KDM5D* is deleted in 52% of human PC cases and more frequently in later stage PC.^11^ Loss of KDM5D has been implicated in chemoresistance in cancer; however, its role in CHK1i sensitivity is unknown.

In this study, we investigated the role of KDM5D in CHK1i sensitivity and elucidated its downstream signaling mechanisms by identifying a novel p38/cyclooxygenase-2 (COX-2) pathway that mediates the effects of KDM5D on CHK1i sensitivity in CRPC and NEPC cells. The identification of KDM5D as a potential regulator of CHK1i sensitivity provides insights into the intrinsic resistance mechanisms of CHK1is and may facilitate the stratification of patients receiving these treatments.

## 2 Materials and methods

### 2.1 Cell culture

LNCaP, C4-2B, 22Rv1, DU145, and NCI-H660 cell lines were obtained from the American Type Culture Collection. Cells were grown in HITE medium (NCI-H660), minimum essential medium supplemented with Eagle’s salt, L-glutamine, RPMI-1640 (LNCaP, C4-2B, 22Rv1), or Ham’s F-12 medium (PC3) supplemented with 10% FBS and penicillin/streptomycin (1×). Parental C4-2B and C4-2B–derived enzalutamide (MDV3100)-resistant, docetaxel-resistant (C4-2B-TaxR), and abiraterone-resistant (C4-2B-AbiR) sublines were obtained from Dr Allen Gao (University of California Davis Medical Center, Sacramento, CA).^13^, ^14^, ^15^ The cultures were maintained at 37 °C in a humidified incubator containing 5% CO_2_. The cells were maintained for <15 passages (<8 days for LNCaP cells).

### 2.2 Real-time IncuCyte cell viability assay

NucLight Red (NR) lentiviral reagent (Sartorius) was used to establish stable expression of nuclear-restricted mKate2 in PC cells to enable efficient and nonperturbing nuclear labeling of live cells for real-time imaging. Stable NR-positive cells were seeded at 5000 to 9000 cells/well in 96-well plates in growth medium containing 10% FBS. Treatments were applied after 24 to 48 hours, and the plates were placed in an IncuCyte S3 Live Cell Imaging System (Sartorius), where real-time images were captured every 6 hours for 4 to 7 days at 10× or 20× magnification throughout the experiment. The red fluorescence signal of the cells was analyzed using the IncuCyte basic or cell-by-cell analyzer module (Sartorius).

### 2.3 Lentiviral expression

The lentiviral vector was generated by cotransfecting 293T cells with the transfer vector TFORF2768 (pLX_TRC317 carrying human *KDM5D*, Addgene plasmid#142159), packaging plasmid psPAX2, and envelope plasmid pMD2.G at a mass ratio of 4:3:1 using PolyJet transfection reagent (SignaGen Laboratories). Lentiviral supernatants were collected at 48 and 72 hours after transfection and filtered through a 0.45 *μ*m filter, and stored at −80 °C. For transduction, PC cells were infected with the lentiviral vector at varying multiplicities of infection in the presence of 8 *μ*g/mL polybrene. After 24 hours, the medium was replenished and the culture was maintained for an additional 48 to 72 hours before the analysis of KDM5D expression. To establish stable cell lines, cells were selected using puromycin (1–2 *μ*g/mL) for 3 days, followed by analysis of gene expression by quantitative real-time reverse transcriptase polymerase chain reaction (RT-qPCR) and Western blotting.

### 2.4 Small interfering RNA transfection

Dicer-substrate small interfering RNAs (DsiRNAs) targeting *KDM5D* or *p38* and nontargeting DsiRNA were obtained from Integrated DNA Technologies. Cells were transfected with siRNAs using Lipofectamine RNAiMAX Transfection Reagent (Invitrogen) according to the manufacturer’s instructions. The DsiRNA sequences are listed in Supplemental Table 1.

### 2.5 Kinase inhibitor screening

A library screening was conducted using the Tocriscreen Kinase Inhibitor Toolbox (#3514, 80 compounds; Tocris Bioscience). 22Rv1-NR cells (10,000 cells/well) in 96-well plates were transfected with *KDM5D* (si-KDM5D) or nontargeting (si-NT) siRNAs for 72 hours. For screening, si-KDM5D-transfected cells were treated with each compound alone (1 *μ*M) or in combination with SRA737 (5 *μ*M; compound + SRA737). Cell viability was monitored over time using the IncuCyte live cell imaging system (Sartorius), with NR fluorescent reporter as a marker for viable cells. The viability ratio (VR) was determined for individual compounds as the ratio of cell viability in compound-treated wells to that in DMSO-treated wells (compound/DMSO). For combination treatments, VR was calculated as the ratio of viability in wells treated with both compound and SRA737 to that in wells treated with SRA737 alone [(compound + SRA737)/SRA737]. The VR measured at 120 hours after treatment was used as the experimental endpoint. Cells transfected with si-NT or si-KDM5D and treated with SRA737 served as controls representing maximum and minimum response levels, respectively.

Hits were identified based on 2 selection criteria: (1) a VR of (compound + SRA737)/SRA737 <1, indicating reversal of si-KDM5D–induced SRA737 resistance; and (2) a VR between 0.8 < (compound/DMSO) <1.2, indicating minimal (<20%) effect on cell viability when administered alone. Compounds meeting both criteria, significantly enhancing SRA737 sensitivity while showing limited single-agent toxicity, were selected for further validation.

### 2.6 Western blotting

Western blotting was performed as described previously.^16^ The cells were lysed in lysis buffer (50 mM Tris-HCl pH 7.5, 150 mM NaCl, 1.5 mM MgCl_2_, 10% glycerol, 1% Triton X-100, 5 mM EGTA, 1 mM Na_3_VO_4_, 10 mM NaF, 1 mM *β*-glycerophosphate, and protease inhibitor cocktail). After sonication, the lysates were centrifuged at maximum speed for 5 minutes, and the amount of protein in the supernatant was quantified using the BCA protein assay kit (Pierce). Cell lysates were subjected to SDS-PAGE, followed by transferring to polyvinylidene difluoride membranes. After preblocking, membranes were incubated with primary antibodies at 4 °C overnight, then washed, and incubated with secondary antibodies at room temperature for 1 hour. An Enhanced Chemiluminescence kit was used to detect the protein bands on the blots (Pierce). The images were captured on an X–ray film, and band intensity was quantified using ImageJ 1.54 software.

### 2.7 Quantitative real-time PCR

RNA extraction was performed on cultured cells using the TRIzol reagent (Thermo Fisher Scientific). RT-qPCR was performed to measure mRNA levels. Total RNA was extracted using TRIzol (Thermo Fisher Scientific) and reverse-transcribed into cDNA using the iScript cDNA synthesis kit (Bio-Rad) according to the manufacturer’s instructions. The resulting cDNA was then amplified using iTaq Universal SYBR Green Supermix (Bio-Rad) on a Bio-Rad CFX Connect Real-Time PCR Detection System. PCR experiments were repeated 3 times with 3 to 6 technical replicates per reaction, using independent culture sets. The relative mRNA expression was normalized to that of a housekeeping gene (glyceraldehyde-3-phosphate dehydrogenase or *β*-actin) and calculated using the 2^-ΔΔCt method. Melting curve analysis was performed to ensure the specificity of amplification. The qPCR primer sequences are listed in Supplemental Table 2.

### 2.8 Bioinformatic analysis

The datasets (GSE184168, GSE128405, GSE116864, and GSE148544) used for generating candidate genes from PC cell lines were acquired from Gene Expression Omnibus (GEO). Patient expression data used to predict drug responses were acquired from Beltran et al.^17^ Drug sensitivity prediction was performed using the pRRophetic package, focusing on solid tumor cell lines.^18^ The pRRophetic package employs ridge regression models trained on pharmacogenomic data from the Cancer Genome Project (2014). Specifically, it uses the gene expression profiles of drug-treated cancer cell lines to build predictive models that estimate IC_50_ values, serving as indicators of patient drug response based on the transcriptomic signatures of individual patient samples.

### 2.9 Statistical analysis

Statistical analysis for cellular data was performed using GraphPad Prism 9.0 software (GraphPad Software, Inc). Continuous outcomes were analyzed using Student’s *t* test for comparison between 2 groups (2-tailed) or ANOVA with Tukey post hoc analysis for ≥3 groups. The correlation between variables was studied using linear regression analysis. Replicate experiments were analyzed both independently and jointly by controlling for batch effects. All values are presented as the mean ± SEM of at least 3 independent experiments. Statistical significance was set at *P <* .05.

## 3 Results

### 3.1 Identification of genes associated with CHK1i sensitivity

**Fig. 1:**
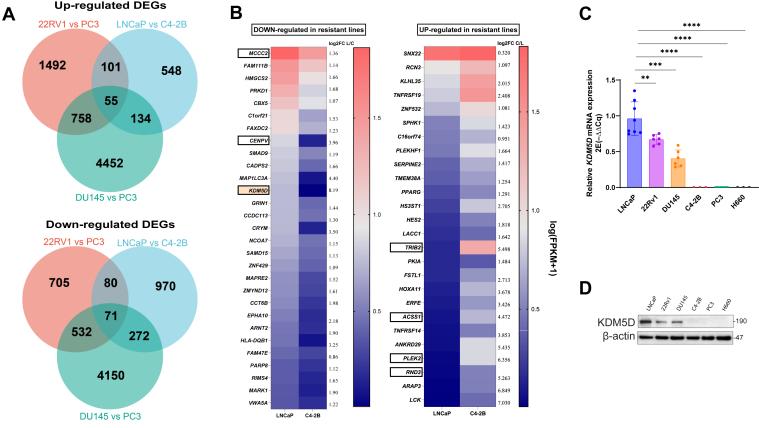
Identifying candidate genes associated with CHK1i sensitivity in PC cells. (A) Venn diagram showing the number of upregulated or downregulated differentially expressed genes (DEGs) from the bioinformatics analysis. (B) Heatmap showing the expression profiles of genes in LNCaP and C4-2B cells that are either downregulated (left) or upregulated (right) in resistant lines. Log_2_ fold change (FC) are shown to the right of the heatmap. Selected candidate genes are outlined. L/C: log_2_FC for LNCaP/C4-2B, C/L: log_2_FC for C4-2B/LNCaP. (C) RT-qPCR validation of *KDM5D* mRNA levels in 6 prostate cancer cell lines. The data are mean ± SEM from 2 experiments, each in triplicate. One-way ANOVA with Kruskal–Wallis for multiple comparisons, ∗∗ *P <* .01, ∗∗∗ *P <* .001, ∗∗∗∗ *P <* .0001. (D) Western blotting analysis of KDM5D expression in PC cells.

CHK1 inhibitors (CHK1i) are an important class of anticancer agents. SRA737 is a new generation of highly selective CHK1i with >1000-fold selectivity against CHK2 and CDK1 (IC_50_: 1.4 nM for CHK1, 2440 nM for CHK2, and 9030 nM for CDK1).^5^^,^^6^ We previously evaluated the sensitivity of a panel of PC cell lines to SRA737 and demonstrated 10- to 20-fold differences in IC_50_s between SRA737-sensitive cells (LNCaP, 22Rv1, DU145) and SRA-resistant PC cells (C4-2B, PC3, NCI-H660).^16^ However, the mechanisms underlying their differential sensitivity to SRA remain unknown. To identify candidate genes that may be responsible for the differential responses to SRA737, gene expression analysis was conducted on these cell lines using data obtained from the Cancer Cell Line Encyclopedia and the GEO. Differentially expressed genes were identified (*P* < .05, |Log_2_ fold change| >1) by comparing sensitive and resistant lines. Specifically, the following comparisons were included: 22Rv1 versus PC3 (Cancer Cell Line Encyclopedia), LNCaP versus C4-2B (GSE184168), and DU145 versus PC3 (GSE128405 and GSE116864). This analysis led to the identification of 126 shared differentially expressed genes, with 55 upregulated and 71 downregulated genes (Fig. 1A). The differentially expressed genes were validated in another GEO dataset that included 4 cell lines, LNCaP, 22Rv1, DU145, and PC3 (GSE148544), followed by crosschecking in 2 isogenic cell lines, LNCaP (sensitive) and C4-2B (resistant) (GSE184186). Only genes that showed a significant fold change >2 (*P* < .05) and relatively higher expression (fragments per kilobase of transcript per million mapped reads >1) in either cell line were selected. The final list of candidate genes that exhibited consistent expression changes were the downregulated (*MCCC2*, *CENPV*, and *KDM5D*) and upregulated (*TRIB2*, *ACSS1*, *PLEK2*, and *RND3*) genes in resistant versus sensitive cell lines (Fig. 1B). Among them, KDM5D was particularly notable, as it was the most downregulated gene in all resistant lines examined (Fig. 1B). The transcript levels of the key candidate genes were further validated by RT-qPCR. *KDM5D* was identified as the only gene consistently downregulated in the resistant versus sensitive lines, whereas this was not observed for another candidate gene, *CENPV* (Supplemental Fig. 1A). This expression pattern of KDM5D was further confirmed at the protein level by Western blot analysis (Fig. 1D). Taken together, our data indicated that KDM5D expression correlated with the sensitivity of CHK1i in PC cells, implying a potential role in regulating CHK1i sensitivity.

### 3.2 KDM5D is required for CHK1i sensitivity in PC cells

**Fig. 2:**
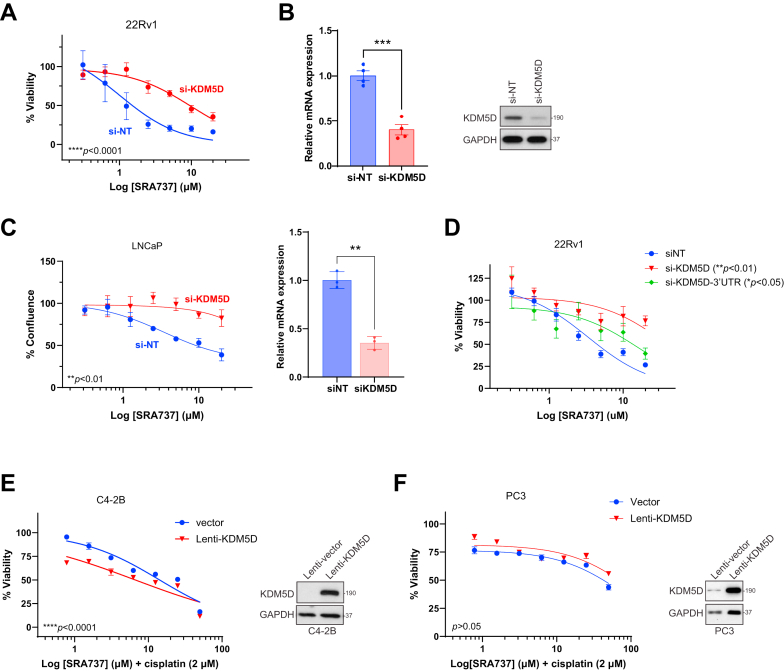
KDM5D was required for SRA737 sensitivity in PC cell lines. (A) Depleting KDM5D caused resistance to SRA737 in 22Rv1 cells. 22Rv1 cells were transfected with nontargeting (si-NT) and KDM5D siRNA (si-KDM5D) for 2 days, followed by SRA737 treatment. Cell viability was monitored by live cell imaging using NR marker. Dose-response curves at 96 hours are shown. Data are from 1 of 3 representative experiments with quadruplicate determinations. ∗∗∗∗*P <* .0001 comparing si-NT to si-KDM5D, two-way ANOVA. (B) Validation of KDM5D knockdown in 22Rv1 by RT-qPCR (left) and Western blot (right). ∗∗∗*P <* .001, *t* test. (C) Depleting KDM5D increased resistance to SRA737 in LNCaP cells. Dose-response curves of LNCaP cells transfected with si-NT and si-KDM5D at 96 hours are shown. ∗∗*P <* .01 comparing si-NT to si-KDM5D, two-way ANOVA. Validation of KDM5D knockdown by RT-qPCR (right). ∗∗*P <* .01, *t* test. (D) Silencing KDM5D by a 3′UTR-targeted siRNA increased resistance to SRA737 in 22Rv1 cells. 22Rv1 cells were similarly transfected for 2 days, followed by SRA737 treatment. Cell viability was monitored by live cell imaging using NR marker. Dose-response curves at 96 hours are shown. Data are from 1 of 3 representative experiments with quadruplicate determinations. ∗∗*P <* .01 comparing si-NT to si-KDM5D, ∗*P <* .05 comparing si-NT to si-KDM5D-3′UTR, two-way ANOVA. (E, F) Overexpression of KDM5D sensitized C4-2B (E) and PC3 cells (F) to SRA737 in the presence of sub-IC_50_ dose of cisplatin. C4-2B or PC3 stably expressing KDM5D (lenti-KDM5D) or empty vector (Vector) were treated with increasing concentrations of SRA737 in the presence of cisplatin (2 *μ*M). KDM5D expression was validated by Western blot (right). Data are from 1 of 3 representative experiments with triplicate determinations. ∗∗∗∗ *P <* .0001 comparing Lenti-KDM5D to vector for C4-2B and not significant (*P* > .05) for PC3, two-way ANOVA.

The causal role of KDM5D in CHK1i sensitivity was further examined by altering the expression of KDM5D in PC cells, followed by the assessment of cell viability after SRA737 treatment. Cell viability was evaluated by real-time live cell imaging using tumor cells stably expressing NR, a fluorescent cell viability marker, as previously described.^16^ For cells with naturally high expression of KDM5D, silencing KDM5D by siRNA led to a nearly 10-fold increase (from 1.4 *μ*M to 12.2 *μ*M, *n* = 3) in IC_50_ to SRA737 in 22Rv1, a SRA737-sensitive cell line (Fig. 2, A and B), demonstrating a key role of *KDM5D* in determining SRA737 sensitivity. Depletion of KDM5D had a small but significant effect on 22Rv1 cell proliferation (Supplemental Fig. 1B), although this effect was unlikely linked to SRA737 sensitivity because the resistance to SRA737 persisted in case of less degree of KDM5D knockdown that did not affect cell growth (data not shown). Similarly, silencing KDM5D caused resistance to SRA737 in LNCaP, another SRA737-sensitive line (IC_50_ from 6.75 to 53.51 *μ*M, *n* = 3) (Fig. 2C). Corroboratively, knocking down KDM5D by an alternative siRNA targeting the 3′UTR region of *KDM5D* also resulted in a significant increase (∼5-fold) in resistance to SRA737 in 22Rv1 cells (Fig. 2D). Thus, loss of KDM5D increased resistance to SRA737 in PC cells.

Next, we examined whether overexpression of KDM5D could sensitize cells resistant to SRA737. Stable clones overexpressing KDM5D were established for resistant cells that had either low (C4-2B) or complete loss (PC3) of KDM5D. Interestingly, overexpression of KDM5D did not increase the sensitivity of these cells to SRA737 nor did it alter their proliferation rates (Supplemental Fig. 2). Thus, although KDM5D is required for SRA737 sensitivity, its overexpression alone is not sufficient to confer sensitivity to SRA737, highlighting the context-dependent role of this gene in CHK1i sensitivity. We sought to increase the basal CHK1 activity in these resistant cells by treating them with a sub-IC_50_ dose of cisplatin (2 *μ*M), a genotoxic agent known to activate CHK1, in order to enhance their sensitivity to SRA737. Interestingly, C4-2B-KDM5D overexpressor exhibited a modest but significant increase in sensitivity to SRA737 compared with the vector control, whereas PC3-KDM5D remained highly resistant to SRA737 (Fig. 2, E and F), reaffirming a cell context-dependent response to SRA737 despite the presence of genotoxic stress.

### 3.3 KDM5D exhibited a broader impact on CHK1i sensitivity and predicted CHK1i resistance in patients with CRPC

**Fig. 3:**
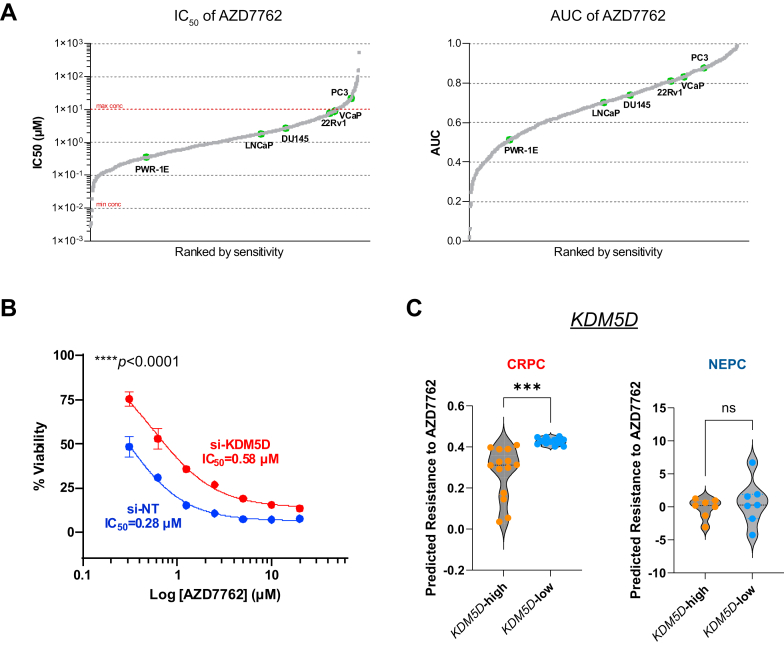
KDM5D predicted patient with CRPC response to CHK1i. (A) IC_50_ and area under the curve (AUC) values for AZD7762 were obtained from GDSC2 dataset and plotted in ascending order of sensitivity across cancer cell lines. Each point represents an individual cell line, with prostate cancer cell lines labeled and highlighted in green. Horizontal dashed red lines in the IC_50_ plot indicate the minimum and maximum concentrations tested in the assay. (B) Silencing KDM5D increased resistance to AZD7762. 22Rv1 cells were transfected with nontargeting (si-NT) and KDM5D siRNA (si-KDM5D) for 2 days, followed by AZD776 treatment. Cell viability was monitored by live cell imaging. ∗∗∗∗*P <* .0001 comparing si-NT to si-KDM5D, two-way ANOVA. (C) High KDM5D predicted increased sensitivity to AZD7762 in patients with CRPC. Predicted IC_50_s to AZD7762 in patients with CRPC (left) or NEPC (right) stratified based on *KDM5D* expression. ∗∗∗*P <* .001, unpaired *t* tests with Welch correction.

To determine whether our findings are applicable to other CHK1is, we examined data related to CHK1i AZD7762 in the Genomics of Drug Sensitivity in Cancer database (GDSC2).^19^ AZD7762 is a potent ATP-competitive CHK inhibitor with equal potency for CHK1 and CHK2 (IC_50_: 5 nM) and has been evaluated in phase 1 clinical trials for multiple cancers.^20^^,^^21^ Based on the GDSC2 data, consistent trends of both IC_50_ and area under the curve were observed across all cancer cell lines, with PC3 displayed higher IC_50_ and area under the curve values, indicative of relatively lower sensitivity to AZD7762 compared with other PC cell lines (DU145, LNCaP, 22Rv1, VCaP, and PWR-1E) (Fig. 3A), which agrees with our observation for SRA737. We further demonstrated that silencing of KDM5D increased the IC_50_ of AZD7762 by 2-fold in 22Rv1 cells (Fig. 3B), implying a broader impact of KDM5D on CHK1i sensitivity in PC cells.

To determine whether our findings are clinically relevant, we stratified patients with CRPC or NEPC based on their expression levels of *KDM5D*^17^ and predicted the potential resistance of these patients to CHK1i AZD7762 using the pRRophetic package.^18^ Patients without predicted values were excluded. The Robust regression and outlier removal method^22^ was used with a *Q*-value of 0.5% to exclude outliers. The predicted IC_50_ of AZD7762 in patients with CRPC (*n* = 26) was lower (more sensitive) for patients with high *KDM5D* than for those with low *KDM5D* (*P <* .001, unpaired *t* tests with Welch correction), further validating *KDM5D*’s role in determining CHK1i sensitivity in CRPC (Fig. 3C). Patients with NEPC (*n* = 14), however, did not exhibit statistically significant differences (*P >* .05), possibly owing to the lower patient numbers. In contrast, another candidate gene, *CENPV* failed to predict the response to AZD7762 in either CRPC or NEPC (Supplemental Fig. 1C). Taken together, these findings indicate a potentially broader effect of KDM5D in response to CHK1i in PC cells and patients.

### 3.4 Elevated KDM5D contributed to increased CHK1i sensitivity in docetaxel-resistant CRPC cells

**Fig. 4:**
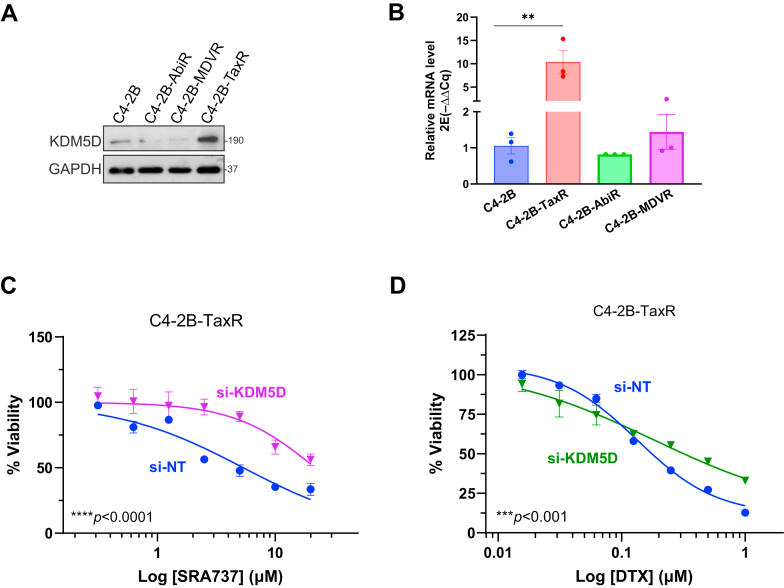
KDM5D accounted for increased CHK1i sensitivity in DTX-resistant CRPC cells. (A) Increase KDM5D expression in DTX-resistant C4-2B-TaxR. Western blot analysis of C4-2B and its drug-resistant sublines. (B) KDM5D is elevated at transcript level in C4-2B-TaxR, measured by RT-qPCR. Data from 1 of 3 representative experiments are shown. ∗∗*P <* .01, *t* test. (C) Depleting KDM5D in C4-2B-TaxR increased resistance to SRA737. Cells were transfected with nontargeting (si-NT) and KDM5D siRNA (si-KDM5D) for 2 days, followed by SRA737 treatment. Cell viability was monitored by live cell imaging, dose-response curves at 96 hours are shown. Data are from 1 of 3 representative experiments with quadruplicate determinations. ∗∗∗∗*P <* .0001 comparing si-NT to si-KDM5D, two-way ANOVA. (D) Knockdown of KDM5D reduced sensitivity of C4-2B-TaxR to DTX. Cells transfected with si-NT or si-KDM5D were treated with increasing doses of DTX. Dose-response curves at 96 h are shown. ∗∗∗*P <* .001 comparing si-NT to si-KDM5D, two-way ANOVA.

We showed previously that C4-2B-TaxR, a docetaxel (DTX)–resistant line derived from C4-2B,^14^ was significantly more sensitive to CHK1i SRA737 (IC_50_: 1.97 ± 1.15 *μ*M for C4-2B-TaxR vs 20.17 ± 5.24 for C4-2B).^16^ C4-2B-TaxR exhibited elevated KDM5D expression at the mRNA and protein levels compared with the parental C4-2B- and C4-2B-derived enzalutamide-resistant (C4-2B-MDVR) and abiraterone-resistant (C4-2B-AbiR) sublines^13^^,^^15^ (Fig. 4, A and B). KDM5D depletion by siRNA resulted in a ∼4-fold increase in resistance to SRA737 in C4-2B-TaxR (*P <* .0001), demonstrating the essential role of KDM5D in sensitizing C4-2B-TaxR to SRA737 (Fig. 4C). In contrast, depletion of KDM5D only modestly enhanced its resistance to DTX (IC_50_s for si-NT and si-KDM5D were 0.13 and 0.18 *μ*M, respectively, *P <* .001), indicating its elevated expression had less effect on DTX resistance developed in this line (Fig. 4D). Note that our IC_50_ for si-NT was comparable to the originally reported IC_50_ value of 0.14 *μ*M for C4-2B-TaxR.^14^ Overall, our findings suggest that DTX-resistant CRPC tumors with elevated KDM5D expression are more responsive to CHK1i treatment.

### 3.5 KDM5D regulated CHK1i sensitivity in part through a p38/COX-2 pathway

**Fig. 5:**
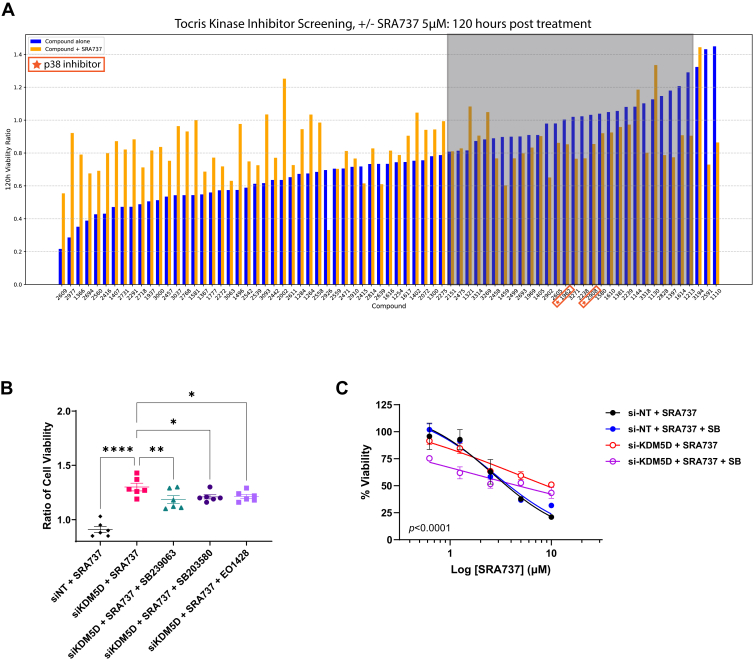
p38 was identified as a mediator of KDM5D-knockdown-induced resistance to SRA737. (A) Identification of p38 inhibitors as potential hits in a library screen. A kinase inhibitor screen was performed on 22Rv1 cells transfected with si-KDM5D. Cells were treated with either the compound alone (1 *μ*M) or in combination with 5 *μ*M SRA (compound + SRA), and cell viability was measured over time. Viability ratios were calculated as compound/DMSO for the inhibitor alone and (compound + SRA)/SRA for the combination treatment. Viability ratio at 120 hours is shown. Cells transfected with si-NT and si-KDM5D and treated with SRA were used as controls. The gray areas indicate the selected hits. The numbers in the red box indicate p38 inhibitors. (B) p38 inhibitors (SB239063, SB203580, and EO1428) at 1 *μ*M partially reversed the KDM5D knockdown-induced resistance to SRA (5 *μ*M). The viability of 22Rv1 cells transfected with si-NT or si-KDM5D and treated with SRA or SRA and p38 inhibitor combinations for 96 hours is shown. ∗*P <* .05, ∗∗*P <* .01, ∗∗∗∗*P <* .0001, *t* test. (C) SB239063 sensitized KDM5D-depleted cells to SRA737. 22Rv1 cells transfected with si-NT or si-KDM5D were treated with increasing concentrations of SRA737 in the presence or absence of SB239063 (5 *μ*M). Data are representative of 3 independent experiments performed in triplicate. ∗∗∗∗*P* < .0001 comparing si-KDM5D + SRA737 to si-KDM5D + SRA737 + SB, two-way ANOVA.

To identify druggable targets involved in KDM5D-regulated CHK1i sensitivity, a kinase inhibitor screen was performed in 22Rv1 cells transfected with si-KDM5D using the Tocris Kinase Inhibitor Library. Hits were defined as compounds that reversed si-KDM5D–induced SRA737 resistance, indicated by a VR of (compound + SRA737)/SRA737 < 1, while exhibiting minimal effects on cell viability when administered alone (a VR between 0.8 < [compound/DMSO] < 1.2). Two structurally distinct p38 inhibitors (SB239063 and EO1428) emerged as likely candidates, as both partially reversed si-KDM5D–induced SRA resistance; however, minimally affected cell viability as single agents (Fig. 5A; Supplemental Table 3). This was validated in subsequent analyses, along with the p38 inhibitor, SB203580 (Fig. 5B). In a subsequent dose-response analysis, SB239063 at 5 *μ*M did not affect the sensitivity of si-NT–transfected control 22Rv1 cells but significantly sensitized KDM5D-knockdown cells to SRA737 (Fig. 5C).

**Fig. 6:**
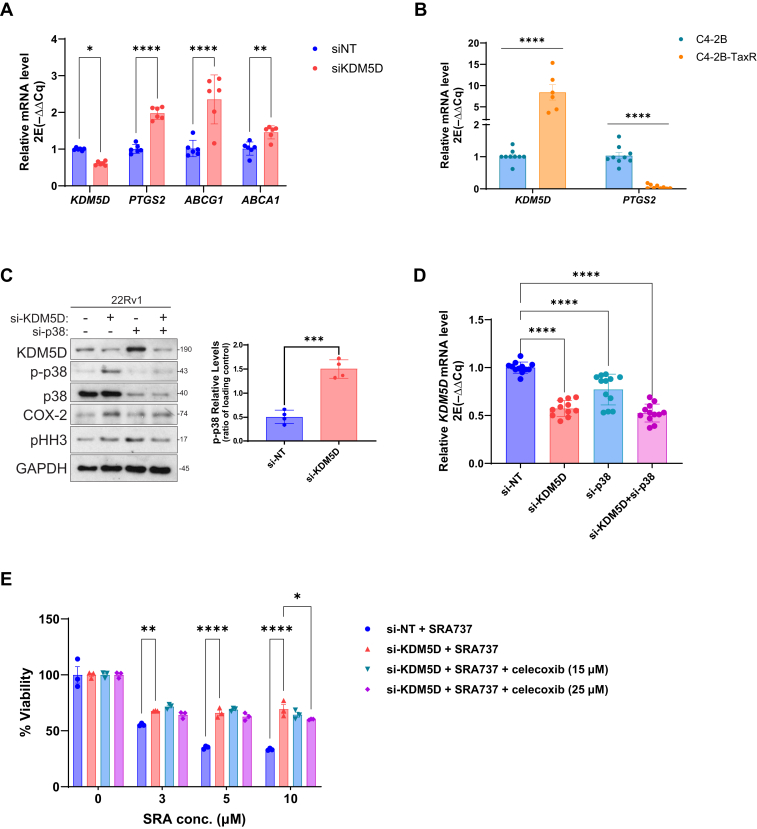
KDM5D modulated CHK1i sensitivity through a p38/COX-2 pathway. (A) Genes induced by silencing KDM5D. Gene expression at mRNA levels was examined by RT-qPCR. (B) *KDM5D* and *PTGS2* mRNAs were examined by RT-qPCR in C4-2B-TaxR cells. (C) Knockdown of KDM5D activated p38, whereas knockdown of p38 upregulated KDM5D. Western blotting analysis of 22Rv1 cells transfected with si-NT, si-KDM5D, si-p38 and the combination of si-KDM5D and si-p38. Right: Quantification of p-p38 was performed by densitometric analysis using ImageJ and normalized to total p38. Data represent mean ± SEM from 3 independent experiments. (D) *KDM5D* transcript levels in 22Rv1 cells transfected as in “C” by RT-qPCR. (E) Inhibition of COX-2 by celecoxib reduced KDM5D-knockdown-induced resistance to SRA (5 *μ*M). 22Rv1 cells transfected with si-NT and si-KDM5D were treated with increasing concentrations of SRA737 in the presence or absence of celecoxib (15, 25 *μ*M). All data shown above are from 1 of 3 independent experiments. For graphs, each data points are from 3 or more replicates. Two-tailed unpaired *t* test were performed for all graphed data. ∗*P* < .05, ∗∗*P* < .005, ∗∗∗*P <* .001, ∗∗∗∗*P* < .0001.

**Fig. 7:**
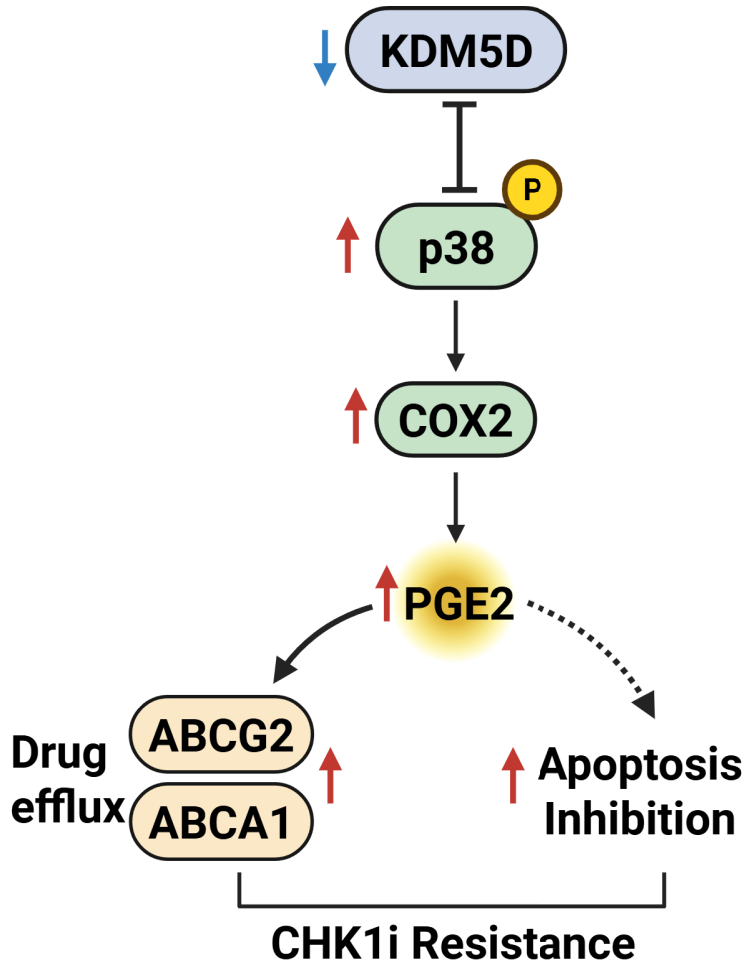
A diagram depicting the mechanism underlying KDM5D-regulated CHK1i sensitivity. In prostate cancer cells, loss of KDM5D activates p38 signaling, which induces COX-2 expression. Elevated COX-2 promotes cell survival by increasing prostaglandin E2 (PGE2) production, leading to upregulation of ABC transporters that may export the CHK1 inhibitor SRA737 and/or by engaging alternative mechanisms that suppress apoptosis. Together, these effects contribute to increased resistance to CHK1 inhibition.

Further analysis showed that *KDM5D* loss led to a ∼2-fold increase in *PTGS2* (COX-2), a well known p38 target gene, and 2 ATP-binding cassette (ABC) transporters, *ABCG2* and *ABCA1*^23^ (Fig. 6A). This agrees with the notion that COX-2 produces prostaglandins, such as prostaglandin E2, which promotes cell survival and could induce drug efflux pumps to cause drug resistance.^24^ Corroboratively, elevated *KDM5D* mRNA levels in C4-2B-TaxR were correlated with low *COX-2* expression (Fig. 6B). Interestingly, depletion of KDM5D increased p38 activity (measured by p-T180/Y182-p38) without affecting p38 protein levels, suggesting that KDM5D depletion–induced p38 activation, but not its expression, drove COX-2 upregulation (Fig. 6C). Silencing of p38 reduced KDM5D-knockdown–induced COX-2 expression, demonstrating a causal role of p38 in COX-2 regulation (Fig. 6C). Additionally, our data showed that silencing p38 reciprocally upregulated KDM5D protein expression, an observation that did not correlate with KDM5D mRNA levels in p38-knockdown cells (Fig. 6D), indicating a posttranscriptional mechanism involved in the regulation of KDM5D expression by p38. Mutual regulation of p38 and KDM5D indicates a negative feedback loop between the 2 genes, which may serve to maintain the homeostatic balance of these important proteins. Finally, similar to p38 inhibitors, celecoxib (a COX-2 inhibitor) reversed KDM5D knockdown-induced SRA resistance, although the effect was only significant at a higher concentration of celecoxib (25 *μ*M) (Fig. 6E). These findings demonstrate a novel role for the p38/COX-2 prosurvival pathway in CHK1i sensitivity, and loss of KDM5D promoted SRA737 resistance in part via p38-driven COX-2 upregulation in CRPC cells, as summarized in the schematic model shown in Fig. 7.

## 4 Discussion

CHK1 is a promising therapeutic target in various cancers. Although potent and selective CHK1is are available and have advanced into clinical trials, heterogeneous responses to these inhibitors remain a major obstacle to their clinical advancement, highlighting the urgent need for predictive biomarkers to guide CHK1i-based treatments. In this study, we identified KDM5D, a male-specific epigenetic regulator, as a key determinant of CHK1i sensitivity in PC cells, with potential predictive value in patients with CRPC. Our data showed that the loss of KDM5D expression correlated with CHK1i resistance. Depletion of KDM5D in KDM5D-expressing PC cells led to a ∼10-fold increase in resistance to SRA737, whereas elevated KDM5D in DTX-resistant CRPC cells contributed to their greater sensitivity to SRA737 relative to the parental line. Loss of KDM5D upregulated COX-2 expression at both the transcript and protein levels via activation of p38, and inhibition of p38 using multiple p38 inhibitors or COX-2 using celecoxib partially reversed the resistance to SRA737 induced by KDM5D knockdown (Fig. 5). Thus, our study identified a novel KDM5D/p38/COX-2 pathway in regulating CHK1i sensitivity in PC cells, whereby reduced KDM5D expression promoted p38/COX-2 activity and increased resistance to CHK1is.

In our recent report, using the TRAMP NEPC mouse model, we demonstrated that CHK1 inhibition by SRA737 potently suppressed tumor growth, metastasis, and recurrence as a single agent, demonstrating its potential for treating advanced PC. Notably, when SRA737 was evaluated across a panel of PC cell lines, a 20-fold variation in sensitivity was observed.^16^ This prompted efforts to identify mediators of differential sensitivity to SRA737 through bioinformatic analysis and subsequent cellular validation, leading to the discovery of KDM5D. Among the cell lines examined, 3 lines (LNCaP, 22Rv1, and DU145) expressed KDM5D transcripts and proteins (Fig. 1, C and D). KDM5D depletion in these cells caused resistance to SRA737, demonstrating the essential role of KDM5D in maintaining SRA737 sensitivity. However, stable overexpression of KDM5D in KDM5D-deficient cells failed to alter their response to SRA737 (Supplemental Fig. 2), indicating that KDM5D alone was not sufficient to restore sensitivity. Thus, additional coregulators are required to complement the effects of KDM5D on CHK1i response. When absent, the resistant phenotype persists despite KDM5D overexpression. This has been demonstrated for other KDM5 family members, such as KDM5C, which requires its interactions with ER*α* and ZMYND8 to activate estrogen target genes to promote breast cancer cell growth.^25^ Interestingly, in the presence of sub-IC_50_ cisplatin, KDM5D-overexpressing C4-2B cells exhibited increased sensitivity to SRA737 relative to the control cells (Fig. 2, D and E), but this was not observed for PC3, another SRA737-resistant line. Cisplatin, a genotoxic agent, induces DNA damage, which activates the Ataxia Telangiectasia and Rad3-related kinase (ATR)/CHK1 pathway to arrest cells for DNA repair.^26^^,^^27^ Our findings suggest that cisplatin-induced DNA damage and subsequent ATR/CHK1 activation could cooperate with overexpressed KDM5D to re-establish SRA737 sensitivity in certain KDM5D-deficient PC cells. In contrast, the study by Komura et al^28^ showed that loss of KDM5D accelerated PC cell proliferation and promoted a more aggressive phenotype, accompanied by increased DNA replication stress and activation of G2/M checkpoint, as evidenced by upregulation of CDK1 and CDC25C and activation of ATR and CHK1. They also found that KDM5D loss sensitized PC cells to ATR inhibitors, highlighting the potential therapeutic value of ATR inhibition in KDM5D-deficient tumors.^28^ Although CHK1 inhibitors were not examined, CHK1 activation (p-CHK1) suggested that these cells might also be more sensitive to CHK1 inhibition. These findings differ from ours, which indicate that loss of KDM5D increases resistance to CHK1 inhibitors. The discrepancies may stem from: (1) Different cell models: Komura et al^28^ primarily used LNCaP and LNCaP-104R2 (a LNCaP-derived CRPC line), whereas we used CRPC 22Rv1 and drug-resistant C4-2B-TaxR cells. Distinct genetic backgrounds may affect responses to loss of KDM5D. (2) Differences in KDM5D knockdown reagents: We observed that si-KDM5D (targeting the coding region) elicited stronger CHK1 resistance than si-KDM5D-3′UTR (targeting the 3′UTR). As KDM5D splice variants have been reported,^29^^,^^30^ different si/shRNAs may variably affect these isoforms, leading to divergent outcomes.

Our data showed elevated KDM5D levels in DTX-resistant C4-2B-TaxR cells relative to the parental and other drug-resistant sublines. It has been reported that KDM5D modulates sensitivity to DTX in PC, where loss of KDM5D induces DTX resistance.^31^ As C4-2B-TaxR was established by prolonged exposure to DTX in culture,^14^ KDM5D could be upregulated to offset the impact of KDM5D loss and may not be functionally important. However, subsequent analysis revealed that KDM5D knockdown converted C4-2B-TaxR from an SRA737-sensitive to a resistant one that resembled the parental line (Fig. 4C), demonstrating that KDM5D upregulation in C4-2B-TaxR contributed to its increased sensitivity to SRA737, corroborating our earlier findings. Compared with the ∼4-fold increase in resistance to SRA737, KDM5D knockdown resulted in only a modest change in resistance to DTX (∼38% increase in IC_50_) (Fig. 4D). This limited effect may reflect a ceiling in the resistance already reached in this DTX-resistant line. Nonetheless, the observed increase in resistance is consistent with a previous report showing that the loss of KDM5D enhances DTX resistance.^31^ Overall, the role of KDM5D in chemoresistance appears to be both cell-context- and agent-dependent, with opposing effects reported for different therapeutic agents. For example, although loss of KDM5D increased DTX resistance in our study, elevated KDM5D expression has been associated with platinum resistance in head and neck squamous cell carcinoma, where KDM5D knockdown restored platinum sensitivity.^32^ Such discrepancies may be due to differences in cancer type, drug class, cellular models, or the nature of resistance, such as acquired (as in the study by Chen et al^32^) versus intrinsic (as in our study and the study by Komura et al^31^). Beyond its role in chemosensitivity, little is known about KDM5D’s involvement in the response to targeted therapies such as CHK1is. Importantly, the increased SRA737 sensitivity observed in DTX-resistant CRPC cells suggests that SRA737 may offer therapeutic benefits for DTX-resistant CRPC tumors, a possibility that warrants further investigation.

Our study identified a novel p38/COX-2 pathway, through which KDM5D regulates CHK1i sensitivity in PC cells. The involvement of p38 was identified through an unbiased kinase inhibitor screen (Fig. 5A), which was validated using multiple p38 inhibitors and p38 siRNA. Our data support the regulation of p38 activity by KDM5D, but not by its expression (Fig. 6C). Notably, a recent report identified KDM5D as a demethylase of p38*α* at K165, and demethylation at K165 inhibited p38*α* and suppressed lung cancer progression.^33^ This agrees with our finding that silencing KDM5D increased p38 activity (measured by p-T180/Y182-p38) in 22Rv1 cells, suggesting that KDM5D may similarly regulate p38 activity in PC cells. p38*α* is abundantly expressed in PC cells and tissues.^34^^,^^35^ Our data also demonstrated a negative feedback loop between KDM5D and p38, where they mutually limit each other’s activity or expression, implying a more complex regulatory circuitry, perhaps to impose homeostatic expression of these genes in cancer cells. The involvement of COX-2 downstream of p38 was confirmed at the transcript, protein, and functional (celecoxib) levels (Fig. 5). This is consistent with COX-2 being a known target of p38^24^^,^^36^^,^^37^ and its role in drug resistance in cancer.^24^ COX-2 produces prostaglandins, such as prostaglandin E2 that promote cell survival and induce drug efflux pumps to cause drug resistance.^24^^,^^38^^,^^39^ Accordingly, we showed that KDM5D silencing significantly upregulated the ABC transporters *ABCG2* and *ABCA1*, indicating the potential role of these ABC transporters in mediating KDM5D loss-induced CHK1i resistance. In addition to its role in regulating ABC transporters, COX-2 modulates several prosurvival pathways such as phosphatidylinositol 3-kinase/protein kinase B, nuclear factor *κ*B, signal transducer and activator of transcription 3, and *β*-catenin, which may contribute to drug resistance. Our data do not exclude the possibility that these pathways are also involved in KDM5D-mediated regulation of CHK1i sensitivity.

In summary, our study identified KDM5D as a novel regulator of CHK1i sensitivity in PC cells, particularly in CRPC, where a high KDM5D predicted a better response to CHK1is. For aggressive tumors with a loss of KDM5D, activation of the prosurvival p38/COX-2 pathway may be exploited as an alternative treatment option. Although additional validation in clinical samples is necessary, our findings highlight KDM5D as a novel potential biomarker for stratifying patients undergoing CHK1i therapy for PC and other malignancies.

## Conflict of interest

Given her role as Editorial Advisory Board at the *Journal of Pharmacology and Experimental Therapeutics*, Q. Jane Wang had no involvement in the peer review of this article and had no access to information regarding its peer review. Full responsibility for the editorial process for this article was delegated to another journal editor. All other authors declare no conflicts of interest.
