# Supplementary material for: Lysine demethylase 5D promotes CHEK1 inhibitor sensitivity through p38-mediated cyclooxygenase-2 expression in castration-resistant prostate cancer cells
Source: J Pharmacol Exp Ther. 2025 Nov 4;392(12):103769. doi: 10.1016/j.jpet.2025.103769 (PMC12799561; doi:10.1016/j.jpet.2025.103769)
Supplement: Supplementary Tables 1-2 and Supplementary Figure 1-2 [file mmc1.docx]

**Supplementary Materials for**

**KDM5D promotes CHK1 inhibitor sensitivity through p38-mediated COX-2 expression in castration-resistant prostate cancer cells**

Wenxiao Zheng, Shichen Li, Raymond Edward West, Ella R. Donahue, Thomas D. Nolin, Song Li, and Q. Jane Wang*

**This file includes:**

Supplemental Table 1

Supplemental Table 2

Supplemental Table 3 (Excel file, legend is included in this file)

Supplemental Figure 1

Supplemental Figure 2

**Supplemental Table 1. List of KDM5D siRNAs used in the study**.

| **siRNA target** | **DsiRNA name** | **RNA sequence 5' to 3' lower case: deoxyribonucleic** |
| --- | --- | --- |
| *KDM5D* | si-KDM5D | sense: GAGGUAAAAGAUAAGGAAUACAAgc |
|  |  | antisense: GCUUGUAUUCCUUAUCUUUUACCUCAU |
| *KDM5D* | si-KDM5D-3'UTR | sense: GUACGUUGUAAUUACCAAAAAGAat |
|  |  | antisense: AUUCUUUUUGGUAAUUACAACGUACAU |

**Supplemental Table 2. List of real time PCR primers used in the study.** F, forward primer; R, reverse primer.

| Target | Primer sequence |
| --- | --- |
| KDM5D | F: 5’-CAAGACCCGCTTGGCTACATT-3’ |
|  | R: 5’-TTGGACGCGAGGAGTAAATCT-3’ |
| CENPV | F: 5’-CCTGCTAGACACCTTTGAATACC-3’ |
|  | R: 5’-TCAGGAGCTTGAAGCGAGAAG-3’ |
| PTGS2 | F: 5’-CTGATGATTGCCCGACTCCC-3’ |
|  | R: 5’-CGCAGTTTACGCTGTCTAGC-3’ |
| ABCA1 | F: 5’-ACCCACCCTATGAACAACATGA-3’ |
|  | R: 5’-GAGTCGGGTAACGGAAACAGG-3’ |
| ABCG2 | F: 5’-TTATCCGTGGTGTGTCTGGA-3’ |
|  | R: 5’-TTCCTGAGGCCAATAAGGTG-3’ |
| ACSS1 | F: 5’-CACAGGACAGACAACAAGGTC-3’ |
|  | R: 5’-CCTGGGTATGGACGATGCC-3’ |
| PLD1 | F: 5’-GAGCCACGGGTAAATACCTCT-3’ |
|  | R: 5’-CCGCGTGTCCAGATTTTCTATG-3’ |
| PLEK2 | F: 5’-GCGATGGTTCATCCTTCGG-3’ |
|  | R: 5’-ATAGCCCCGGTGATCTCAAAG-3’ |
| RND3 | F: 5’-GCTCCATGTCTTCGCCAAG-3’ |
|  | R: 5’-AAAACTGGCCGTGTAATTCTCA-3’ |
| NTNG1 | F: 5’-GCCCTTTGGGTTACGGTGT-3’ |
|  | R: 5’-CCCTTCTTCCGTGTAAATCTGAG-3’ |
| MCCC2 | F: 5’-AAAGCCCGAGCACTTCACATA-3’ |
|  | R: 5’-TCCAATGCCTGTAATAATGCCAC-3’ |
| TRIB2 | F: 5’-ATGAACATACACAGGTCTACCCC-3’ |
|  | R: 5’-GGGCTGAAACTCTGGCTGG-3’ |

**Supplemental Table 3. Viability ratio (VR) data from kinase inhibitor screen.** 22Rv1-NR cells (10,000 cells/well) in 96-well plates were transfected with *KDM5D* (si-KDM5D) or non-targeting (si-NT) siRNAs for 72 h. For screening, si-KDM5D-transfected cells were treated with each compound alone (1 μM) or in combination with SRA737 (5 μM; compound + SRA737). VR was determined for individual compounds as the ratio of cell viability in compound-treated wells to that in DMSO-treated wells (compound/DMSO). For combination treatments, VR was calculated as the ratio of viability in wells treated with both compound and SRA to that in wells treated with SRA alone [(compound + SRA737)/SRA737]. The VR measured at 120 hours post-treatment was used as the experimental endpoint. Cells transfected with si-NT or si-KDM5D and treated with SRA737 served as controls representing maximum and minimum response levels, Cell viability was monitored over time using the IncuCyte live cell imaging system (Sartorius), with a NucLight Lentivirus Red (NR) fluorescent reporter as a marker for viable cells.


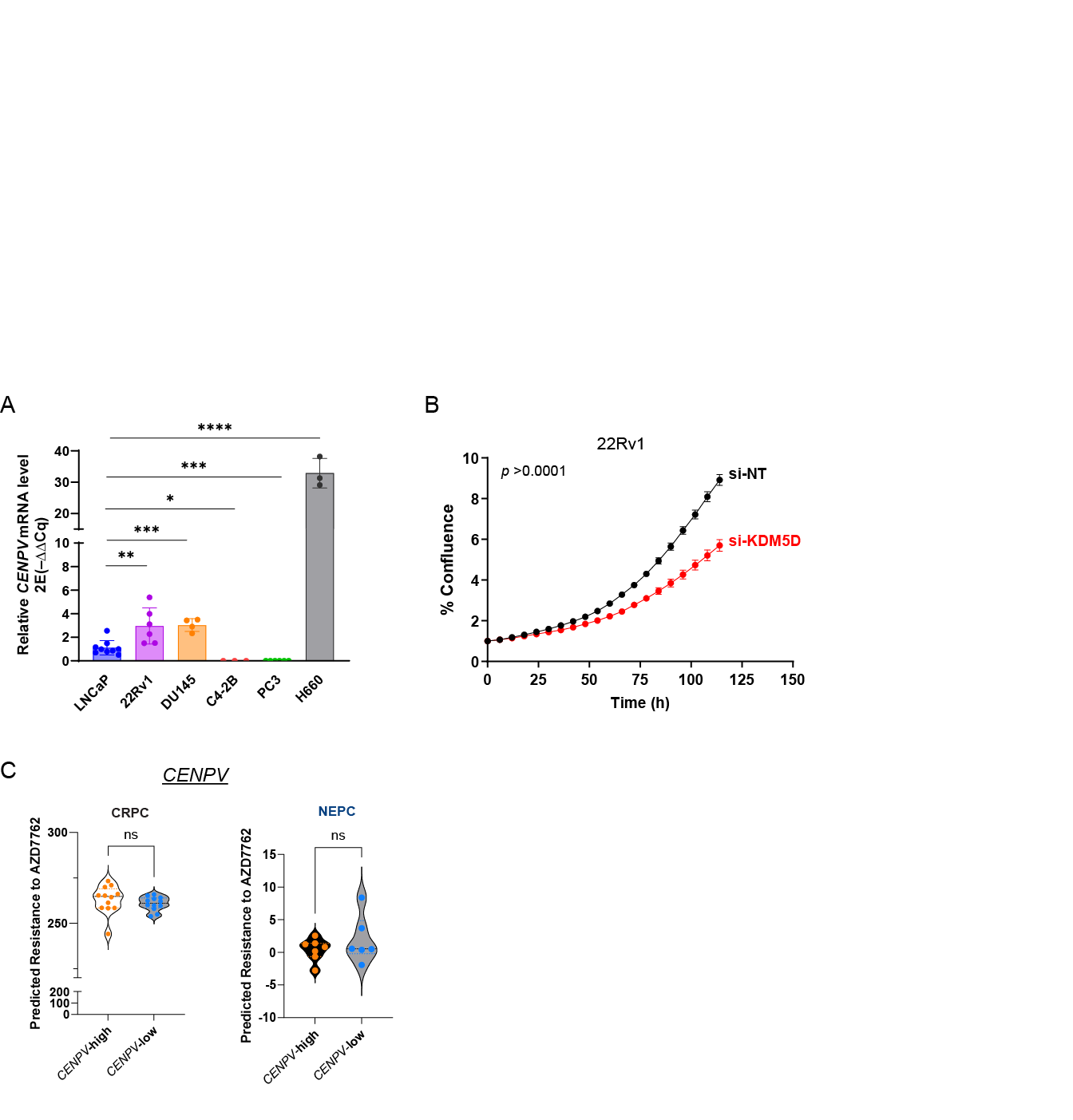


**Supplemental Figure 1.** (**A**) RT-qPCR analysis of CENPV mRNA levels in PC cell lines. Two-tailed unpaired *t*-test. * *p <* 0.05, ** *p <* 0.005, *** *p <* 0.0005. (**B**) Depleting KDM5D did not significantly impact 22Rv1 cell proliferation. Proliferation of 22Rv1 transfected with si-NT and si-KDM5D was measured over time. *p >* 0.05, two-way ANOVA. (**C**) *CENPV* levels did not predict sensitivity to AZD7762 in CRPC and NEPC patients. Predicted IC50s to AZD7762 in CRPC (n=24, *left*) or NEPC (n=13, *right*) patients stratified based on *CENPV* expression. ns, *p >* 0.05, unpaired t-tests with Welch’s correction.


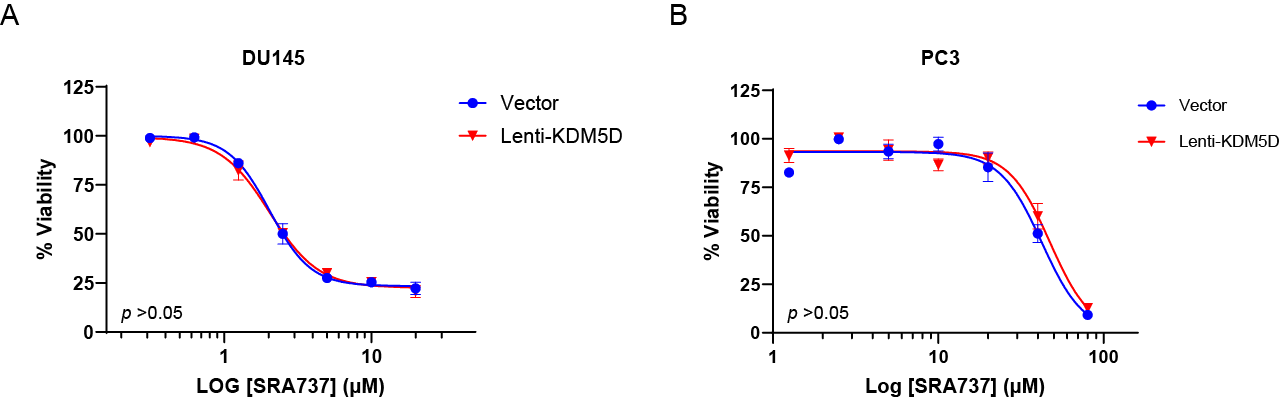


**Supplemental Figure 2. O**verexpressing KDM5D did not increase the sensitivity of DU145 (A) or PC3 (B) cells to SRA737, nor did it alter their rates of proliferation.
